# Is the MDS-UPDRS a Good Screening Tool for Detecting Sleep Problems and Daytime Sleepiness in Parkinson's Disease?

**DOI:** 10.1155/2014/806169

**Published:** 2014-11-17

**Authors:** Krisztina Horváth, Zsuzsanna Aschermann, Péter Ács, Edit Bosnyák, Gabriella Deli, Endre Pál, József Janszky, Béla Faludi, Ildikó Késmárki, Sámuel Komoly, Magdolna Bokor, Eszter Rigó, Júlia Lajtos, Péter Klivényi, György Dibó, László Vécsei, Annamária Takáts, Adrián Tóth, Piroska Imre, Ferenc Nagy, Mihály Herceg, Anita Kamondi, Eszter Hidasi, Norbert Kovács

**Affiliations:** ^1^Doctoral School of Clinical Neuroscience, University of Pécs, Rét Utca 2, Pécs 7623, Hungary; ^2^Department of Neurology, University of Pécs, Rét Utca 2, Pécs 7633, Hungary; ^3^MTA-PTE Clinical Neuroimaging Research Group, Rét Utca 2, Pécs 7623, Hungary; ^4^Unified Health Centers of the City of Pécs, Dr. Veress Endre Utca 2, Pécs 7633, Hungary; ^5^Department of Neurology, Nyirő Gyula Hospital-National Institute of Psychiatry and Addictology, Dévai Utca 15a, Budapest, Hungary; ^6^Department of Neurology, Kenézy Gyula Hospital, Bartók Béla Utca 2-26, Debrecen, Hungary; ^7^Department of Neurology, University of Szeged, Semmelweis Utca 6, Szeged, Hungary; ^8^MTA-SZTE Neuroscience Research Group, Semmelweis Utca 6, Szeged, Hungary; ^9^Department of Neurology, Semmelweis University, Balassa Utca 6, Budapest, Hungary; ^10^Department of Neurology, Csolnoky Ferenc Hospital, Kórház Utca 1, Veszprém, Hungary; ^11^Department of Neurology, Kaposi Mór Hospital, Tallián Gyula Utca 20-32, Kaposvár 7400, Hungary; ^12^National Institute of Clinical Neuroscience, Amerikai Utca 57, Budapest, Hungary; ^13^Department of Neurology, University of Debrecen, Móricz Zsigmond Körút 22, Debrecen, Hungary

## Abstract

Movement Disorder Society-sponsored Unified Parkinson's Disease Rating Scale (MDS-UPDRS) has separate items for measuring sleep problems (item 1.7) and daytime sleepiness (1.8). The aim of our study was to evaluate the screening sensitivity and specificity of these items to the PD Sleep Scale 2nd version (PDSS-2) and Epworth Sleepiness Scale (ESS). In this nationwide, cross-sectional study 460 PD patients were enrolled. Spearman's rank correlation coefficients were calculated between the individual items, domains, and the total score of PDSS-2 and item 1.7 of MDS-UPDRS. Similarly, the items and the total score of ESS were contrasted to item 1.8 of MDS-UPDRS. After developing generalized ordinal logistic regression models, the transformed and observed scores were compared by Lin's Concordance Correlation Coefficient. Only item 3 difficulties staying asleep and the “disturbed sleep” domain of PDSS-2 showed high correlation with “sleep problems” item 1.7 of the MDS-UPDRS. Total score of PDSS-2 had moderate correlation with this MDS-UPRDS item. The total score of ESS showed the strongest, but still moderate, correlation with “daytime sleepiness” item 1.8 of MDS-UPDRS. As intended, the MDS-UPDRS serves as an effective screening tool for both sleep problems and daytime sleepiness and identifies subjects whose disabilities need further investigation.

## 1. Introduction 

Recently the nonmotor symptoms (NMS) of Parkinson's disease (PD) have been increasingly recognized as major burden of quality of life [1, 2]. The recently published Movement Disorder Society-sponsored Unified Parkinson's Disease Rating Scale (MDS-UPDRS) was developed to assess a wide variety of nonmotor symptoms with good scale properties. However, there are only a few published studies available contrasting the MDS-UPDRS to other validated scales on NMS. One of the papers demonstrated that the MDS-UPDRS Part I for nonmotor experiences of daily living had a strong convergent validity to the Non-Motor Symptoms Questionnaire with the exception of patients with the most severe NMS [1]. However, the NMS Questionnaire is a general scale and not an instrument specially designed for certain aspects of PD. In another recently published paper, the individual items of MDS-UPDRS Part I were validated against a number of scales for assessing individual NMS. However, this study included a relatively low number of patients (*n* = 94) and utilized the sole means of correlation calculations [3]. Although this research confirmed that the MDS-UPDRS Part I total score had a strong convergent validity with a composite score of scales for the NMS of PD and the majority of items of MDS-UPDRS Part I had at least moderate correlation with the representative nonmotor scales, the methodology applied was unable to confirm agreement and concordance. In our opinion, Spearman's correlation coefficient is an inappropriate measure of reliability because the strength of linear association, and not agreement, is measured. Therefore, it is possible to have a high degree of correlation even if agreement is poor [4, 5]. Because one of the most important questions in assessing the model validity is if the model-predicted values are precise and accurate at the same time [6], concordance correlation measurements should be utilized to establish agreement (concordance) by two scales.

Sleep-related problems are one of the most important and troublesome nonmotor aspects of PD. These problems are certainly multidimensional. For example, sleep-disturbances associated with PD might be equally due to troublesome nighttime OFF symptoms and other issues not specific for PD (e.g., microarousals caused by sleep apnea syndrome). Similarly, daytime sleepiness in PD might also be caused by PD-specific or nonspecific causes.

MDS-UPDRS has separate items for measuring nighttime sleep-related problems (item 1.7) and daytime sleepiness (1.8) each ranging from 0 (not present) to 4 (severe) scores. The aim of our study was to evaluate the relationship of these items to the PD Sleep Scale 2nd version (PDSS-2) and the Epworth Sleepiness Scale (ESS).

The Parkinson's Disease Sleep Scale 2nd version is a validated scale to assess sleep problems in PD [7]. It is composed of 15 items evaluating three domains. Each item has a 5-point Likert-type scale ranging from 0 “never” to 4 “very often” (except for item 1 which is reversed). Each domain consists of clusters of five questions (motor symptoms at night: 4, 5, 6, 12, and 13; PD symptoms at night: 7, 9–11, and 15; and disturbed sleep: 1–3, 8, and 14) [7]. Symptoms on each domain can be scored in the range of 0–20 points, higher scores meaning more nocturnal disturbance. The sum of the three domains gives the total score of PDSS-2 with the maximum value of 60 points. The threshold indicating sleep problems is 11 points for the Hungarian version of PDSS-2 [8–10].

Epworth Sleepiness Scale is composed of 8 items investigating a single domain to assess daytime sleepiness. The usefulness of ESS was demonstrated in PD by several studies [11–14]. Each item is scaled from 0 “would never doze” to 3 “high chance to doze.” The total score of ESS is the sum of all item scores (0–24 points), higher scores representing more severe daytime sleepiness. The cutoff value indicating daytime sleepiness in Hungarian PD patients is 5 points [8].

## 2. Materials and Methods 

### 2.1. Patients

In this nationwide cross-sectional multicenter study 460 patients fulfilling the UK Brain Bank criteria for PD were enrolled. Each subject gave written consent in accordance with the ethical approval of the National Ethical Committee (184/2013. 14437/2013/EKU). Each patient was examined by a neurologist specialized in movement disorders. A portion of these patients (357/460) participated in the program of cultural adaptation and validation of the MDS-UPDRS into Hungarian. Data were summarized at University of Pécs (by KH and NK). Subsequently, levodopa equivalent dosage calculations were performed [15].

Patients were screened for the presence of dementia by the means of the Hungarian validated versions of Mattis Dementia Rating Scale [16] (MDRS, *n* = 345 patients) and the Montreal Cognitive Assessment [17] (MOCA, *n* = 460 patients). Patients receiving scores ≤125 points on Mattis test and/or scores ≤22 points on MOCA were considered as having major neurocognitive disorders [16].

### 2.2. Obtained Rating Scales

Besides sociodemographic and PD-historic data, the Hungarian validated versions of MDS-UPDRS [18], PDSS-2 [8], and ESS [8, 19] were obtained. Because data from these scales were categorical, nonparametric tests were applied. As being part of the MDS-UPDRS, Hoehn-Yahr Scale was also used to detect the overall severity of PD. For description, medians with interquartile range (IR, 25th–75th percentiles) were calculated.

### 2.3. Prevalence and Correlations

Because the score of 0 on the items means symptom-free condition, the prevalence of each item was based on the portion of subjects having the score >0 point on that particular item.

For correlation, Spearman's rank correlation coefficients were calculated. For correlation coefficients, the values 0–0.299 were indicative of weak correlation, 0.300–0.599 were indicative of moderate correlation, and 0.600–1.000 were considered as high association [20].

### 2.4. Regression Models and Lin's Concordance Correlation

To evaluate the relationship between the scales, models of generalized ordinal logistic regression were built with bootstrap replication. Because most regression algorithms are very sample specific, their generalizability to other samples is quite limited. We chose a generalized logistic regression model with lasso regularization as this method allowed for bootstrap cross validation. Using this option we could generalize the results to samples outside the index sample.

To determine the relationship between the PDSS-2 and the “sleep problems” item of MDS-UPDRS (1.7), generalized ordinal logistic regression was applied. First, we converted the PDSS-2 scores to MDS-UPDRS item 1.7 by regression analysis. Subsequently, Lin's Concordance Correlation Coefficient (LCCC) [21] was used to calculate the concordance between the true MDS-UPDRS scores and those estimated from the PDSS-2. The concordance was categorized by the value of LCCC as acceptable (0.601–0.900) and good (0.901–1.000).

Similar approach was utilized to determine the relationship between ESS and “daytime sleepiness” item of MDS-UPDRS (1.8). Based on the obtained generalized ordinal logistic regression model we converted the ESS scores to MDS-UPDRS item 1.8 and subsequently LCCC were calculated between the true and estimated MDS-UPDRS 1.8 values.

### 2.5. Receiver Operating Characteristic (ROC) Curve

In order to analyze how efficiently the MDS-UPDRS differentiates patients having sleep-related problems from those who do not have any sleep-related problems, we applied ROC analysis. After categorization of patients into two groups based on their total score on PDDS-2 (patients with sleep problems: total PDSS-2 score ≥11 points and patients without sleep problems: <11 points), we performed a second categorization based on MDS-UPDRS item 1.7 score (patients with sleep problems: >0 points, patients without sleep problems: 0 points). Subsequently these two different categorization methods were compared to obtain positive and negative predictive values, specificity, and sensitivity.

Afterwards, we also evaluated how appropriately the MDS-UPDRS 1.8 item identified patients with daytime sleepiness. Categorization based on the ESS scores (patients having daytime sleepiness: total score ≥5 points versus those not having daytime sleepiness, ESS <5 points) was compared to that of MDS-UPDRS item 1.8.

### 2.6. Statistical Analysis

All statistical analyses were carried out using IBM SPSS software package (version 21, SPSS Inc., Chicago, USA). Statistical significance level was set to 5%. Because the SPSS Suite did not have built-in functions for calculating LCCC, the freely available syntax files developed by Marta Garcia-Granero were utilized (available: http://gjyp.nl/marta/, accessed at Jan 7, 2013). Positive and negative predictive values were calculated by the utilization of the syntax adapted from the IBM website (http://www-01.ibm.com/support/docview.wss?uid=swg21483380).

## 3. Results 

### 3.1. Demographic and PD-Related Clinical Data

The subject population consisted of 460 PD patients. The clinical characteristics are demonstrated in Table 1. The LED was 214 mg (14–324 mg, median and 25th–75th percentiles).

### 3.2. Sleep Problems

Based on MDS-UPDRS item 1.7, 315 patients (68.4%) had sleep-related problems (prevalence, score >0). Only 1.7% of patients had the total score of 0 on PDSS-2. According to the PDSS total score, 324 patients (70.4%) had sleep-related problems clinically (score ≥11 points). Item 8 had the highest prevalence on PDSS-2 (nocturia: 91.0%), while the lowest was at item 7 (hallucinations: 15.0%). Out of the three domains of PDSS-2, the “disturbed sleep” had the highest prevalence (98.3%). Prevalence, frequency, and median values of sleep-related items are demonstrated in Table 2.

Eight items of PDSS-2 had poor correlation with the “sleep problems” item of MDS-UPDRS (6: distressing dreams at night, 7: nighttime distressing hallucinations, 8: nocturia, 9: immobility at night, 11: nighttime muscle cramps in extremities, 12: painful posturing in the morning, 13: tremor, and 15: snoring or difficulties in breathing). Six items had moderate correlation with item 1.7 of MDS-UPDRS (1: bad sleep quality, 2: difficulties falling asleep, 4: restlessness of extremities at night, 5: urge to move extremities, 10: pain in extremities, and 14: tired and sleepy after waking in the morning). Furthermore, the total score and two domains of PDSS-2 (motor symptoms and PD symptoms at night) had also moderate correlation coefficients. Only a single item (3: difficulties staying asleep) and the “disturbed sleep” domain of PDSS-2 had high correlation with the “sleep problems” item of MDS-UPDRS (Table 2).

Generalized ordinal logistic regression model was developed for PDSS-2 items to estimate MDS-UPDRS item 1.7. Only PDSS items 1, 2, 3, and 5 contributed significantly to the model (Supplementary Table 1 in Supplementary Materials available online at http://dx.doi.org/10.1155/2014/806169). The details of the ordinal logistic regression model are presented in Supplementary Table 2. The predicted MDS-UPDRS 1.7 values were subsequently compared to the true MDS-UPDRS 1.7 values by LCCC. The value of LCCC was 0.833 (95% confidence interval: 0.809–0.864) demonstrating acceptable agreement between the PDSS-2 and the “sleep problems” item of MDS-UPDRS.

Based on the ROC analysis, MDS-UPDRS item 1.7 can differentiate between patients with and without sleep-related problems measured by PDSS-2 reliably (positive predictive value: 0.714 with the 95% CI of 0.633–0.794; negative predictive value: 0.851 with the 95% CI of 0.809–0.887; sensitivity: 0.754 with the 95% CI of 0.672–0.831; and specificity: 0.827 with 95% CI: 0.783–0.866).

### 3.3. Daytime Sleepiness

Based on MDS-UPDRS item 1.8, 345 patients (75.0%) had problems with daytime sleepiness (prevalence, score >0). Only 4.74% of patients had the total score of 0 points on ESS. According to ESS total score, 312 patients (67.8%) clinically had daytime sleepiness (ESS score ≥5 points). None of the items of ESS showed high correlation with MDS-UPDRS item 1.8. While items 1–4 had moderate correlation, items 5–8 had only poor correlation. The highest value of correlation coefficient was observed between the total score of ESS and item 1.8 of MDS-UPDRS (0.494) (Table 3).

Generalized ordinal logistic regression model was developed for ESS items to estimate MDS-UPDRS item 1.8. Only ESS items 1, 2, 3, and 7 contributed significantly to the model (Supplementary Table 3). The details of the ordinal logistic regression model are presented in Supplementary Table 4. The predicted MDS-UPDRS 1.8 values were subsequently compared to the true MDS-UPDRS 1.8 values by LCCC. The value of LCCC was 0.638 (95% confidence interval: 0.561–0.708) demonstrating acceptable agreement between ESS and “daytime sleepiness” item of MDS-UPDRS.

Based on the ROC analysis, MDS-UPDRS item 1.8 can differentiate between patients with and without daytime sleepiness measured by ESS reliably (positive predictive value: 0.617 with the 95% CI of 0.527–0.703; negative predictive value: 0.777 with the 95% CI of 0.731–0.819; sensitivity: 0.580 with the 95% CI of 0.500–0.660; and specificity: 0.859 with 95% CI: 0.817–0.895).

## 4. Discussion 

Our data supports that the PDSS-2 has a strong and convergent validity to “sleep problems” item of MDS-UPDRS. Several items of disturbed sleep domain (items 1–3) had the best correlation with MDS-UPDRS item 1.7. This relationship is not surprising, because the question of item 1.7 queries the following: “Over the past week, have you had trouble going to sleep at night or staying asleep through the night? Consider how rested you felt after waking up in the morning.” The possible answers in MDS-UPDRS emphasize the length of staying asleep (1: easily able to get a full night sleep, 2: with difficulties can achieve a full night sleep, 3: stay asleep more than the half the night, and 4: less than half of the night). The most concordant item (number 3) of PDSS-2 asks also about difficulty staying asleep, and the other acceptably concordant questions (1: bad sleep quality and 2: difficulties falling asleep) also contribute to this “disturbed sleep” domain.

For daytime sleepiness, we found considerably smaller correlation between the ESS and MDS-UPDRS item 1.8 than Gallagher and coworkers did [3]. In their paper Spearman's correlation coefficient was 0.56 based on the sample of 94 patients while our 0.494 value was based on the analysis of 460 patients. The discrepancy might be due to the differences in the sample size and other patient characteristics. We might assume at least two reasons behind these low correlation coefficients: MDS-UPDRS and ESS apply different time-frame and severity measurements. Whereas ESS asks the patient to base his/her answer referring to “recent times,” the MDS-UPDRS assesses the usual function “over the past week including today.” ESS evaluates how likely the patient will doze off in different situations (e.g., sitting and reading, being a passenger in a car, and sitting and talking to someone else). This type of severity scale assesses the frequency and likelihood to differentiate between scores 1–3. On the contrary, the severity scale of MDS-UPDRS item 1.8 is based not only on the frequency (e.g., 3: sometimes versus 4: often), but also on the different types of conditions where the patient would fall asleep (e.g., 2: being alone and relaxing; 3-4: eating or talking to people). Despite these concerns, we found acceptable agreement between the ESS items and the daytime sleepiness item of MDS-UPDRS.

The authors are aware of some limitations of the study. Despite having a relatively large sample of patients, the majority of the subjects had mild to moderate disease severity (Hoehn-Yahr Scale: 1–3). To overcome this problem, we utilized a generalized logistic regression model with lasso regularization as this method allowed for bootstrap cross validation. Using this option we could generalize the results to samples outside the index sample.

## 5. Conclusions

Our data demonstrate that the MDS-UPDRS nonmotor experiences of daily works as a good screening questionnaire aimed at identifying areas of disability related to sleep problems and daytime sleepiness. The scale itself registers presence and severity of items but has an official APPENDIX with recommended more specific scales to pursue areas in more detail. As a screening tool, there are some aspects of sleep and daytime sleepiness which are not covered by MDS-UPDRS, but the presence of nonzero scores on these MDS-UPDRS items allows for fruitful utilization of more specific and detailed rating instruments. In our view, studies focusing on the sleep-related problems in PD can begin with the MDS-UPDRS to accurately identify the cohort with sleep problems, and then this group can be studied with the PDDS-2 and ESS serving to assess all major dimensions in greater detail.

## Supplementary Material

Supplementary data: This files detailed information on the developedgeneralized ordinal logistic models.

## Figures and Tables

**Table 1:** Clinical characteristics of the study cohort.

Characteristics	Mean/number	Percentage
Age	65.6 ± 9.7	
Disease-duration	8.3 ± 7.8	
Education years	11.8 ± 3.2	
Sex	293 males	63.7%
Fluctuation	236	51.3%
Dyskinesia	184	40.0%
Hoehn-Yahr stage I	31	6.7%
Hoehn-Yahr stage II	275	59.8%
Hoehn-Yahr stage III	115	25.0%
Hoehn-Yahr stage IV	28	6.1%
Hoehn-Yahr stage V	11	2.4%
Deep brain stimulation therapy	113	24.5%
Levodopa/carbidopa intestinal gel infusion therapy	10	2.2%
Levodopa	358	77.8%
Dopamine agonists	299	65.0%
Without medication	14	3.0%
Levodopa and dopamine agonist combination	118	25.7%
COMT-inhibition	208	45.2%
MAO-inhibition	108	23.5%
Anticholinergic therapy	10	2.2%
Presence of dementia	**10**	**2.2%**
Antipsychotic medication	**8**	**1.74%**
Sedative medication usage	**25**	**5.43%**

Presence of dementia was defined as having scores ≤125 points on Mattis Dementia Rating Scale and/or scores ≤22 points on Montreal Cognitive Assessment.

**Table 2:** Prevalence, frequency, and correlation values for sleep-related problems.

	Prevalence	Frequency of score 0	Frequency of score 1	Frequency of score 2	Frequency of score 3	Frequency of score 4	Median	Percentile 25	Percentile 75	Correlation with MDS-UPDRS item 1.7
MDS-UPDRS item 1.7	68.5%	145	100	82	108	25	1	0	3	1.000

PDSS item number 01	68.0%	147	125	99	58	31	1	0	2	.573^**^
PDSS item number 02	50.9%	226	79	91	44	20	1	0	2	.546^**^
PDSS item number 03	67.4%	150	87	81	73	69	1	0	3	.682^**^
PDSS item number 04	52.2%	220	74	86	53	27	1	0	2	.369^**^
PDSS item number 05	51.1%	225	80	80	48	27	1	0	2	.390^**^
PDSS item number 06	43.0%	262	91	79	18	10	0	0	1	.156^**^
PDSS item number 07	15.0%	391	40	19	5	5	0	0	0	.169^**^
PDSS item number 08	91.1%	41	97	102	109	111	2	1	3	.275^**^
PDSS item number 09	48.7%	236	61	64	62	37	0	0	2	.238^**^
PDSS item number 10	51.3%	224	74	86	50	26	1	0	2	.323^**^
PDSS item number 11	57.2%	197	101	94	45	23	1	0	2	.283^**^
PDSS item number 12	42.8%	263	68	62	45	22	0	0	2	.268^**^
PDSS item number 13	45.7%	250	64	60	54	32	0	0	2	.127^**^
PDSS item number 14	70.9%	134	135	102	56	33	1	0	2	.302^**^
PDSS item number 15	31.7%	314	65	56	15	10	0	0	1	.189^**^

PDSS motor domain	82.7%						4	2	7	.398^**^
PDSS PD symptoms domain	82.3%						3	1	6	.358^**^
PDSS disturbed sleep domain	97.0%						8	4	11	.707^**^
PDSS total score	98.3%						15	9	23	.598^**^

^**^ *P* < 0.01.

**Table 3:** Prevalence, frequency, and correlation values for daytime sleep-related problems.

	Prevalence	Frequency of score 0	Frequency of score 1	Frequency of score 2	Frequency of score 3	Frequency of score 4	Median	Percentile 25	Percentile 75	Correlation with MDS-UPDRS item 1.8
MDS-UPDRS item 1.8	75.1%	115	88	224	29	5	2	1	2	1.000

ESS item number 01	69.6%	140	161	109	51		1	0	2	.423^**^
ESS item number 02	82.2%	82	157	150	72		1	1	2	.446^**^
ESS item number 03	42.1%	267	122	45	27		0	0	1	.373^**^
ESS item number 04	51.8%	222	130	79	30		1	0	1	.330^**^
ESS item number 05	88.5%	53	103	114	191		2	1	3	.293^**^
ESS item number 06	15.4%	390	50	20	1		0	0	0	.249^**^
ESS item number 07	55.5%	205	129	76	51		1	0	2	.278^**^
ESS item number 08	13.9%	397	45	17	2		0	0	0	.254^**^

ESS total score	95.3%						7	4	10	.494^**^

^**^ *P* < 0.01.

## Abbreviations

ESS: Epworth Sleepiness Scale
LCCC: Lin’s Concordance Correlation Coefficient
MDRS: Mattis Dementia Rating Scale
MDS-UPDRS: Movement Disorder Society-sponsored Unified Parkinson’s Disease Rating Scale
MOCA: Montreal Cognitive Assessment
NMS: Nonmotor symptoms
PD: Idiopathic Parkinson’s disease
PDSS-2: Parkinson’s Disease Sleep Scale 2nd version
ROC: Receiver Operating Characteristic.
