# Supplementary material for: Is the MDS-UPDRS a Good Screening Tool for Detecting Sleep Problems and Daytime Sleepiness in Parkinson's Disease?
Source: Parkinsons Dis. 2014 Nov 17;2014:806169. doi: 10.1155/2014/806169 (PMC4251816; doi:10.1155/2014/806169)
Supplement: Supplementary file 1 — Supplementary data: This files detailed information on the developedgeneralized ordinal logistic models. [file 806169.f1.pdf]

Supplementary Table1. Tests of model effects for the generalized ordinal logistic models evaluating the sleep related problems item of MDS-UPDRS.

Tests of Model Effects

Source	Type III		
	Wald Chi-Square	df	Sig.
PDSS_01	35,643	4	,000
PDSS_02	25,506	4	,000
PDSS_03	81,840	4	,000
PDSS_04	1,580	4	,812
PDSS_05	10,528	4	,032
PDSS_06	2,532	4	,639
PDSS_07	6,080	4	,193
PDSS_08	7,001	4	,136
PDSS_09	1,552	4	,817
PDSS_10	2,917	4	,572
PDSS_11	2,179	4	,703
PDSS_12	,344	4	,987
PDSS_13	5,135	4	,274
PDSS_14	5,604	4	,231
PDSS_15	2,961	4	,564

Dependent Variable: @1_7

Model: (Threshold), PDSS_01, PDSS_02, PDSS_03, PDSS_04, PDSS_05, PDSS_06, PDSS_07, PDSS_08, PDSS_09, PDSS_10, PDSS_11, PDSS_12, PDSS_13, PDSS_14, PDSS_15

Supplementary Table 2. Model parameters for the generalized ordinal logistic models evaluating the sleep related problems item of MDS-UPDRS

Parameter Estimates

Parameter		B	Std. Error	95% Wald Confidence Interval		Hypothesis Test			Exp(B)	95% Wald Confidence Interval for Exp(B)	
				Lower	Upper	Wald Chi-Square	df	Sig.		Lower	Upper
Threshold	[@1_7=4]	-8,190	,5659	-9,299	-7,081	209,409	1	,000	,000	9,152E-005	,001
	[@1_7=3]	-4,938	,4625	-5,844	-4,031	113,958	1	,000	,007	,003	,018
	[@1_7=2]	-3,349	,4321	-4,196	-2,502	60,081	1	,000	,035	,015	,082
	[@1_7=1]	-1,504	,4009	-2,290	-,718	14,078	1	,000	,222	,101	,488
[PDSS_01=4]		-1,695	,4604	-2,597	-,793	13,556	1	,000	,184	,074	,453
[PDSS_01=3]		-2,059	,4069	-2,857	-1,262	25,619	1	,000	,128	,057	,283
[PDSS_01=2]		-1,710	,3422	-2,381	-1,040	24,989	1	,000	,181	,092	,354
[PDSS_01=1]		-,945	,2927	-1,519	-,372	10,428	1	,001	,389	,219	,690
[PDSS_01=0]		0[a]	.	.	.	.	.	.	1	.	.
[PDSS_02=4]		-1,884	,5634	-2,988	-,779	11,178	1	,001	,152	,050	,459
[PDSS_02=3]		-1,973	,4358	-2,827	-1,119	20,494	1	,000	,139	,059	,327
[PDSS_02=2]		-,625	,3095	-1,231	-,018	4,073	1	,044	,535	,292	,982
[PDSS_02=1]		-,621	,2978	-1,205	-,038	4,355	1	,037	,537	,300	,963
[PDSS_02=0]		0[a]	.	.	.	.	.	.	1	.	.
[PDSS_03=4]		-3,362	,4168	-4,179	-2,545	65,056	1	,000	,035	,015	,078
[PDSS_03=3]		-2,440	,3877	-3,200	-1,680	39,606	1	,000	,087	,041	,186
[PDSS_03=2]		-2,025	,3289	-2,670	-1,381	37,920	1	,000	,132	,069	,251
[PDSS_03=1]		-,843	,3250	-1,480	-,207	6,736	1	,009	,430	,228	,813
[PDSS_03=0]		0[a]	.	.	.	.	.	.	1	.	.
[PDSS_04=4]		-,016	,6079	-1,207	1,176	,001	1	,979	,984	,299	3,241
[PDSS_04=3]		,379	,4275	-,459	1,217	,786	1	,375	1,461	,632	3,377
[PDSS_04=2]		,021	,3190	-,605	,646	,004	1	,948	1,021	,546	1,908
[PDSS_04=1]		-,170	,3177	-,793	,453	,286	1	,593	,844	,453	1,573
[PDSS_04=0]		0[a]	.	.	.	.	.	.	1	.	.
[PDSS_05=4]		-1,480	,6581	-2,770	-,190	5,059	1	,024	,228	,063	,827
[PDSS_05=3]		-1,118	,4325	-1,965	-,270	6,675	1	,010	,327	,140	,764
[PDSS_05=2]		-,509	,3304	-1,156	,139	2,371	1	,124	,601	,315	1,149
[PDSS_05=1]		,058	,3149	-,559	,676	,034	1	,853	1,060	,572	1,965
[PDSS_05=0]		0[a]	.	.	.	.	.	.	1	.	.
[PDSS_06=4]		-,051	,7832	-1,586	1,484	,004	1	,948	,950	,205	4,411
[PDSS_06=3]		-,377	,5707	-1,496	,741	,437	1	,509	,686	,224	2,099
[PDSS_06=2]		,015	,3013	-,575	,606	,003	1	,959	1,015	,563	1,833
[PDSS_06=1]		,367	,2732	-,168	,903	1,807	1	,179	1,444	,845	2,466
[PDSS_06=0]		0[a]	.	.	.	.	.	.	1	.	.
[PDSS_07=4]		,171	1,0676	-1,921	2,264	,026	1	,873	1,187	,146	9,620
[PDSS_07=3]		-,992	1,0119	-2,975	,991	,961	1	,327	,371	,051	2,694

[PDSS_07=2]	-,957	,5304	-1,997	,083	3,256	1	,071	,384	,136	1,086
[PDSS_07=1]	-,639	,3801	-1,384	,107	2,822	1	,093	,528	,251	1,112
[PDSS_07=0]	0[a]	.	.	.	.	.	.	1	.	.
[PDSS_08=4]	,353	,4358	-,502	1,207	,654	1	,419	1,423	,606	3,342
[PDSS_08=3]	-,230	,4347	-1,082	,622	,281	1	,596	,794	,339	1,862
[PDSS_08=2]	-,003	,4275	-,840	,835	,000	1	,995	,997	,431	2,306
[PDSS_08=1]	,474	,4373	-,383	1,332	1,177	1	,278	1,607	,682	3,787
[PDSS_08=0]	0[a]	.	.	.	.	.	.	1	.	.
[PDSS_09=4]	-,089	,4238	-,920	,741	,045	1	,833	,914	,398	2,098
[PDSS_09=3]	,184	,3358	-,474	,842	,301	1	,584	1,202	,622	2,322
[PDSS_09=2]	-,246	,3177	-,869	,376	,602	1	,438	,782	,419	1,457
[PDSS_09=1]	-,198	,3193	-,824	,427	,386	1	,534	,820	,439	1,533
[PDSS_09=0]	0[a]	.	.	.	.	.	.	1	.	.
[PDSS_10=4]	,566	,6965	-,799	1,931	,661	1	,416	1,762	,450	6,898
[PDSS_10=3]	,237	,4282	-,603	1,076	,306	1	,580	1,267	,547	2,933
[PDSS_10=2]	,367	,3240	-,268	1,002	1,284	1	,257	1,444	,765	2,725
[PDSS_10=1]	,466	,3125	-,146	1,078	2,224	1	,136	1,594	,864	2,940
[PDSS_10=0]	0[a]	.	.	.	.	.	.	1	.	.
[PDSS_11=4]	-,811	,6205	-2,027	,405	1,708	1	,191	,444	,132	1,500
[PDSS_11=3]	-,335	,4161	-1,151	,481	,648	1	,421	,715	,316	1,617
[PDSS_11=2]	-,049	,3167	-,670	,572	,024	1	,877	,952	,512	1,771
[PDSS_11=1]	-,122	,2772	-,666	,421	,194	1	,659	,885	,514	1,524
[PDSS_11=0]	0[a]	.	.	.	.	.	.	1	.	.
[PDSS_12=4]	-,269	,5552	-1,357	,819	,235	1	,628	,764	,257	2,268
[PDSS_12=3]	,047	,4304	-,797	,891	,012	1	,913	1,048	,451	2,437
[PDSS_12=2]	,020	,3361	-,639	,679	,003	1	,953	1,020	,528	1,971
[PDSS_12=1]	,007	,3170	-,614	,629	,001	1	,982	1,007	,541	1,875
[PDSS_12=0]	0[a]	.	.	.	.	.	.	1	.	.
[PDSS_13=4]	-,075	,4240	-,906	,756	,031	1	,859	,928	,404	2,129
[PDSS_13=3]	,374	,3388	-,290	1,038	1,218	1	,270	1,453	,748	2,824
[PDSS_13=2]	,476	,3304	-,172	1,124	2,074	1	,150	1,609	,842	3,076
[PDSS_13=1]	,550	,3129	-,063	1,163	3,091	1	,079	1,733	,939	3,201
[PDSS_13=0]	0[a]	.	.	.	.	.	.	1	.	.
[PDSS_14=4]	-,087	,5075	-1,082	,907	,030	1	,863	,916	,339	2,477
[PDSS_14=3]	-,717	,3850	-1,471	,038	3,466	1	,063	,488	,230	1,038
[PDSS_14=2]	-,383	,2974	-,966	,200	1,658	1	,198	,682	,381	1,221
[PDSS_14=1]	-,526	,2728	-1,060	,009	3,714	1	,054	,591	,346	1,009
[PDSS_14=0]	0[a]	.	.	.	.	.	.	1	.	.
[PDSS_15=4]	,646	,8230	-,967	2,259	,615	1	,433	1,907	,380	9,570
[PDSS_15=3]	-,369	,5861	-1,518	,780	,396	1	,529	,691	,219	2,181
[PDSS_15=2]	,052	,3462	-,626	,731	,023	1	,881	1,053	,534	2,076
[PDSS_15=1]	,407	,3031	-,187	1,001	1,800	1	,180	1,502	,829	2,721
[PDSS_15=0]	0[a]	.	.	.	.	.	.	1	.	.
(Scale)	1[b]									

Dependent Variable: @1_7

Model: (Threshold), PDSS_01, PDSS_02, PDSS_03, PDSS_04, PDSS_05, PDSS_06, PDSS_07, PDSS_08, PDSS_09, PDSS_10, PDSS_11, PDSS_12, PDSS_13, PDSS_14, PDSS_15

a. Set to zero because this parameter is redundant.

b. Fixed at the displayed value.

Supplementary Table3. Tests of model effects for the generalized ordinal logistic models evaluating the daytime sleepiness item of MDS-UPDRS.

Tests of Model Effects

Source	Type III		
	Wald Chi-Square	df	Sig.
ESS_01	10,106	3	,018
ESS_02	24,574	3	,000
ESS_03	8,747	3	,033
ESS_04	5,247	3	,155
ESS_05	4,032	3	,258
ESS_06	2,729	2	,256
ESS_07	8,683	3	,034
ESS_08	5,084	3	,166

Dependent Variable: @1_8

Model: (Threshold), ESS_01, ESS_02, ESS_03, ESS_04, ESS_05, ESS_06, ESS_07, ESS_08

Supplementary Table 4. Model parameters for the generalized ordinal logistic models evaluating the daytimesleepiness item of MDS-UPDRS

Parameter Estimates

Parameter		B	Std. Error	95% Wald Confidence Interval Lower	95% Wald Confidence Interval Upper	Hypothesis Test Wald Chi-Square	Hypothesis Test df	Hypothesis Test Sig.	Exp(B)	95% Wald Confidence Interval for Exp(B) Lower	95% Wald Confidence Interval for Exp(B) Upper
Threshold	[@1_8=4]	-7,690	,6070	-8,879	-6,500	160,474	1	,000	,000	,000	,002
	[@1_8=3]	-5,424	,4159	-6,239	-4,609	170,088	1	,000	,004	,002	,010
	[@1_8=2]	-1,710	,3217	-2,341	-1,080	28,271	1	,000	,181	,096	,340
	[@1_8=1]	-,587	,3096	-1,194	,019	3,599	1	,058	,556	,303	1,020
[ESS_01=3]		-1,036	,4453	-1,908	-,163	5,408	1	,020	,355	,148	,850
[ESS_01=2]		-1,012	,3361	-1,671	-,353	9,059	1	,003	,364	,188	,703
[ESS_01=1]		-,403	,2492	-,891	,086	2,610	1	,106	,669	,410	1,090
[ESS_01=0]		0[a]	.	.	.	.	.	.	1	.	.
[ESS_02=3]		-2,017	,4272	-2,854	-1,180	22,293	1	,000	,133	,058	,307
[ESS_02=2]		-1,218	,3302	-1,865	-,571	13,601	1	,000	,296	,155	,565
[ESS_02=1]		-1,031	,2881	-1,596	-,467	12,814	1	,000	,357	,203	,627
[ESS_02=0]		0[a]	.	.	.	.	.	.	1	.	.
[ESS_03=3]		-,677	,5087	-1,674	,320	1,772	1	,183	,508	,187	1,377
[ESS_03=2]		-,550	,3953	-1,325	,224	1,939	1	,164	,577	,266	1,252
[ESS_03=1]		-,681	,2362	-1,144	-,218	8,309	1	,004	,506	,319	,804
[ESS_03=0]		0[a]	.	.	.	.	.	.	1	.	.
[ESS_04=3]		-,572	,4777	-1,508	,364	1,434	1	,231	,564	,221	1,440
[ESS_04=2]		-,608	,3254	-1,246	,030	3,493	1	,062	,544	,288	1,030
[ESS_04=1]		,077	,2421	-,397	,552	,102	1	,749	1,081	,672	1,737
[ESS_04=0]		0[a]	.	.	.	.	.	.	1	.	.
[ESS_05=3]		-,370	,3461	-1,048	,308	1,142	1	,285	,691	,351	1,361
[ESS_05=2]		,121	,3623	-,589	,831	,112	1	,738	1,129	,555	2,296
[ESS_05=1]		-,101	,3474	-,782	,580	,084	1	,771	,904	,458	1,786
[ESS_05=0]		0[a]	.	.	.	.	.	.	1	.	.
[ESS_06=2]		-,844	,5411	-1,905	,216	2,434	1	,119	,430	,149	1,241
[ESS_06=1]		,052	,3464	-,627	,731	,022	1	,881	1,053	,534	2,077
[ESS_06=0]		0[a]	.	.	.	.	.	.	1	.	.
[ESS_07=3]		-,506	,3897	-1,270	,258	1,684	1	,194	,603	,281	1,295
[ESS_07=2]		,577	,3279	-,065	1,220	3,101	1	,078	1,781	,937	3,388
[ESS_07=1]		,319	,2422	-,156	,794	1,732	1	,188	1,375	,856	2,211
[ESS_07=0]		0[a]	.	.	.	.	.	.	1	.	.
[ESS_08=3]		1,040	1,2947	-1,497	3,577	,645	1	,422	2,829	,224	35,782
[ESS_08=2]		-1,014	,5744	-2,139	,112	3,114	1	,078	,363	,118	1,119
[ESS_08=1]		-,474	,3677	-1,195	,246	1,664	1	,197	,622	,303	1,279
[ESS_08=0]		0[a]	.	.	.	.	.	.	1	.	.
(Scale)		1[b]									

Dependent Variable: @1_8

Model: (Threshold), ESS_01, ESS_02, ESS_03, ESS_04, ESS_05, ESS_06, ESS_07, ESS_08

a. Set to zero because this parameter is redundant.

b. Fixed at the displayed value.
